# *Strychnos pseudoquina* and Its Purified Compounds Present an Effective *In Vitro* Antileishmanial Activity

**DOI:** 10.1155/2013/304354

**Published:** 2013-09-30

**Authors:** Paula Sousa Lage, Pedro Henrique Rocha de Andrade, Amanda de Santana Lopes, Miguel Angel Chávez Fumagalli, Diogo Garcia Valadares, Mariana Costa Duarte, Daniela Pagliara Lage, Lourena Emanuele Costa, Vivian Tamietti Martins, Tatiana Gomes Ribeiro, José Dias de Souza Filho, Carlos Alberto Pereira Tavares, Rodrigo Maia de Pádua, João Paulo Viana Leite, Eduardo Antonio Ferraz Coelho

**Affiliations:** ^1^Programa de Pós-Graduação em Ciências da Saúde: Infectologia e Medicina Tropical, Faculdade de Medicina, Universidade Federal de Minas Gerais, Belo Horizonte, Minas Gerais 31.270-901, Brazil; ^2^Departamento de Patologia Clínica, COLTEC, Universidade Federal de Minas Gerais, Belo Horizonte, Minas Gerais 31.270-901, Brazil; ^3^Departamento de Bioquímica e Biologia Molecular, Universidade Federal de Viçosa, Viçosa, Minas Gerais 36.570-000, Brazil; ^4^Departamento de Bioquímica e Imunologia, Instituto de Ciências Biológicas, Universidade Federal de Minas Gerais, Belo Horizonte, Minas Gerais 31.270-901, Brazil; ^5^Programa de Pós-Graduação em Ciências Farmacêuticas, Faculdade de Farmácia, Universidade Federal de Minas Gerais, Belo Horizonte, Minas Gerais 31.270-901, Brazil; ^6^Departamento de Química, Universidade Federal de Minas Gerais, Belo Horizonte, Minas Gerais 31.270-901, Brazil; ^7^Departamento de Produtos Farmacêuticos, Faculdade de Farmácia, Universidade Federal de Minas Gerais, Belo Horizonte, Minas Gerais 31.270-901, Brazil

## Abstract

The development of new and cost-effective alternative therapeutic strategies to treat leishmaniasis has become a high priority. In the present study, the antileishmanial activity of *Strychnos pseudoquina* St. Hil. was investigated and pure compounds that presented this biological effect were isolated. An ethyl acetate extract was prepared, and it proved to be effective against *Leishmania amazonensis*. A bioactivity-guided fractionation was performed, and two flavonoids were identified, quercetin 3-O-methyl ether and strychnobiflavone, which presented an effective antileishmanial activity against *L. amazonensis*, and studies were extended to establish their minimum inhibitory concentrations (IC_50_), their leishmanicidal effects on the intra-macrophage *Leishmania* stage, as well as their cytotoxic effects on murine macrophages (CC_50_), and in O_+_ human red blood cells. The data presented in this study showed the potential of an ethyl acetate extract of *S. pseudoquina*, as well as two flavonoids purified from it, which can be used as a therapeutic alternative on its own, or in association with other drugs, to treat disease evoked by *L. amazonensis*.

## 1. Introduction

Leishmaniasis is a disease caused by the protozoa of the *Leishmania* genus. The parasites are transmitted through the bite of an infected sandfly, and more than 20 *Leishmania* species are responsible for diseases in humans [1]. Leishmaniasis is endemic in 98 countries throughout Africa, Asia, Southern Europe, and Latin America. The disease is also emergent in dogs living in the United States, Canada, Northern Italy, and Germany [2–4]. The impact of the disease on human health has been grossly underestimated for many years, and the World Health Organization has classified leishmaniasis as one of the six most important neglected tropical diseases [2]. 

The treatment of leishmaniasis has been based on the use of pentavalent antimonials; however, increased parasite resistance and side effects, such as arthralgias, myalgias, pancreatitis, leukopenia, and cardiotoxicity, are important problems reported by patients [5–7]. Liposomal amphotericin B (AmpB) is considered effective, though these formulations are very expensive [8]. In addition, leishmaniasis has emerged as an opportunistic infection in human immunodeficiency virus-infected patients [9]; therefore, the development of new and cost-effective alternative therapeutic strategies to treat the disease has become a priority [10].

In recent years, considerable attention has been given to secondary compounds purified from plants in an attempt to search for new antileishmanial drugs [6, 11–13]. Although studies employing extracts and/or purified molecules presenting antileishmanial activity have been undertaken, and to date, no effective and alternative products have been formulated that can be applied to threat leishmaniasis.

The *Strychnos* genus includes approximately 200 plant species, many of which are known for their potential medicinal secondary metabolites [14, 15]. *Strychnos pseudoquina* St. Hil. is a native cinchona-like tree of the Brazilian savanna, popularly known as “quina” and used in the folk medicine to treat hepatic and stomach diseases [16], fever, and malaria [17]. Phytochemical and biological studies employing *S. pseudoquina* have demonstrated the presence of some alkaloids and flavonoids that present antiplasmodial and/or antitumoral activity [18–20]. In the case of flavonoids, results have indicated their pharmacological activity and potential benefit to general human health [21]. Due to the popularity of *S. pseudoquina* as a medicinal plant, the present study was developed to evaluate the antileishmanial activity of an ethyl acetate extract obtained from this plant in an attempt to purify compounds responsible for this biological activity using a bioactivity-guided fractionation. Two flavonoids were isolated, quercetin 3-O-methyl ether and strychnobiflavone, which presented an effective antileishmanial activity against *Leishmania amazonensis*, and studies were extended to establish their minimum inhibitory concentrations (IC_50_), their leishmanicidal effects on the intra-macrophage *Leishmania* stage, as well as their cytotoxic effects on murine macrophages (CC_50_), and in O_+_ human red blood cells.

## 2. Materials and Methods

### 2.1. Chemicals and General Details

Reagents and solvents were obtained from commercial sources and were used as derived. Column chromatography was carried out using silica gel F254 (230–400 mesh) as a stationary phase. Thin layer chromatography (TLC) was carried out using aluminum sheets precoated with silica gel 60 F254 (Merck). 1D and 2D NMR experiments were performed on a Bruker AVANCE DRX400 and DPX200 spectrometers at the Department of Chemistry, Federal University of Minas (UFMG), Brazil, in CD_3_OD or DMSO-*d*_6_ at 300 K, using tetramethylsilane (TMS) as the chemical shift internal standard for both nuclei. UPLC-MS/MS analyses were carried out using an ACQUITY Ultra Performance LC system (Waters, Milford, MA, USA) linked simultaneously to both PDA 2996 photo diode array detector (Waters, Milford, MA, USA) and an ACQUITY TQ Detector (Waters MS Technologies, Manchester, UK), equipped with a Z-spray electrospray ionization (ESI) source operating in positive mode. MassLynx software (version 4.1, Waters, Milford, MA, USA) was used to control the instruments, as well as for data acquisition and processing.

### 2.2. Plant Material and Identification

*Strychnos pseudoquina* St. Hil. stem bark was collected in a Brazilian savanna region, in the district of Uberlândia (Uberlândia, Minas Gerais, Brazil). A voucher specimen was deposited in the Herbarium of the Federal University of Uberlândia (UFU) (code HUFU 10936). The vegetable material was selected and air-dried at room temperature for 1 week. 

### 2.3. Bioactivity-Guided Fractionation and Purification

Exactly 480 g of the pulverized stem barks was submitted to percolation with hexane, and the material was sequentially submitted to exhaustive percolation with ethyl acetate at room temperature. The solvent was removed by evaporation to yield the ethyl acetate extract (AESP, 36.30 g, 7.6%). Later, the extract was subjected to silica gel column chromatography and eluted in different gradients of dichloromethane-ethyl acetate followed by ethyl acetate-ethanol 98%, with a progressive increase in the polarity of the mobile phase, providing 456 fractions. Fractions that shown similar TLC data were combined, formulating 29 different groups, which were evaluated in their antileishmanial activity in such a way as to select the groups that presented the best antileishmanial activity and to identify the pure compounds responsible for this biological activity.

### 2.4. Parasites and Mice

*L. amazonensis* (IFLA/BR/1967/PH-8) was used. Parasites were grown at 24°C in Schneider's medium (Sigma, St. Louis, MO, USA), supplemented with 20% heat-inactivated fetal bovine serum (FBS, Sigma), 20 mM L-glutamine, 200 U/mL penicillin, and 100 *μ*g/mL streptomycin, at pH 7.4. Stationary-phase promastigotes were prepared as described [23]. Murine peritoneal macrophages were obtained from female BALB/c mice (8 weeks old), which were purchased from the Institute of Biological Sciences of UFMG. The Animal Use Committee from UFMG approved the experimental protocol (code 136/2012).

### 2.5. Antileishmanial Activity

The inhibition of *Leishmania* growth was assessed *in vitro* by cultivating stationary-phase promastigotes of *L. amazonensis* (1 × 10^6^ cells) in the presence of ethyl acetate extract (0.78 to 100 *μ*g/mL), 29 purified fractions (50 *μ*g/mL, each one), quercetin 3-O-methyl ether (1.09 to 140 *μ*M) or strychnobiflavone (0.62 to 79.5 *μ*M), in 96-well culture plates (Nunc, Nunclon, Roskilde, Denmark) for 48 h at 24°C. A previous titration curve was performed to determine the best time of inhibition of *L. amazonensis* growth incubating the evaluated products (data not shown). Cell viability was assessed by measuring the cleavage of 2 mg/mL of MTT [3-(4.5-dimethylthiazol-2-yl)-2.5-diphenyl tetrazolium bromide] (Sigma). Absorbances were measured by using a multiwell scanning spectrophotometer (LABTRADE, model 660) at a wavelength of 570 nm. Amphotericin B (1 *μ*M) was used as a positive control. The concentration of the products needed to inhibit 50% of the *Leishmania* viability (IC_50_) was determined by applying the sigmoidal regression of the concentration-response curves, using different concentrations of the compounds. Data shown are representative of four independent experiments, performed in triplicate, which presented similar results.

### 2.6. Cytotoxicity Assay and Hemolytic Activity

The inhibition of 50% of the macrophage viability (CC_50_) was calculated by cultivating macrophages (5 × 10^5^ cells) with ethyl acetate extract (10 to 50 *μ*g/mL), quercetin 3-O-methyl ether (1.09 to 140 *μ*M), or strychnobiflavone (0.62 to 79.5 *μ*M), in 96-well plates for 48 h at 37°C. A previous titration curve was performed to determine the best time of inhibition of macrophages viability incubating the evaluated products (data not shown). Cell viability was assessed by the MTT assay, and amphotericin B was used as a control. The selectivity index (SI) of the products was calculated by determining the ratio between IC_50_ and CC_50_. The hemolytic activity was investigated by incubating the products, in the same concentrations used for cytotoxicity evaluation, with a 5% red blood cell (human O^+^) suspension for 1 h at 37°C. Briefly, the erythrocyte suspension was centrifuged (1000 ×g for 10 min), and cell lysis was determined spectrophotometrically (570 nm), as described [24]. The absence of (blank) or 100% presence of hemolysis (positive control) was determined by replacing the products for an equal volume of PBS or distilled water, respectively. The results were determined by the percentage of hemolysis when compared with the negative and positive controls. Data shown are representative of four independent experiments, performed in triplicate, which presented similar results. 

### 2.7. Inhibition of Infection in Phagocytic Cells

The inhibitory effect of quercetin 3-O-methyl ether and strychnobiflavone on the *Leishmania* invasion of macrophages was evaluated using promastigotes of* L. amazonensis*. Parasites (1 × 10^6^ cells) were preincubated with different concentrations of quercetin 3-O-methyl ether (0, 70, 140, or 280 *μ*M) or strychnobiflavone (0, 40, 80, or 160 *μ*M) for 4 h at 24°C. A previous titration curve was performed to determine the minimum time incubation of parasites with the compounds, in order to obtain the maximum of inhibition of infection into the host cells (data not shown). After incubation, parasites were washed three times with RPMI 1640 medium, quantified, and incubated for 24 h with murine macrophages, using a ratio of 10 parasites per 1 macrophage. After, cells were washed, set, and stained to determine the percentage of infected macrophages by counting 200 cells in triplicate. Data shown are representative of four independent experiments, performed in triplicate, which presented similar results.

### 2.8. Treatment of Infected Macrophages

Murine macrophages (5 × 10^5^ cells) were plated on round glass coverslips inside the wells of a 24-well culture plate (Nunc) in an RPMI 1640 medium supplemented with 20% FBS, 2 mM L-glutamine, 200 U/mL penicillin, and 100 *μ*g/mL streptomycin, at pH 7.4. After 24 h of incubation at 37°C in 5% CO_2_, stationary-phase promastigotes of *L. amazonensis *were added to the wells (5 × 10^6^ cells), and the cultures were incubated for 24 h at 37°C in 5% CO_2_. A previous titration curve was performed to determine the minimum time incubation of parasites with the macrophages, in order to obtain the maximum of infection into the host cells (data not shown). Next, free parasites were removed by extensive washing with an RPMI 1640 medium, and infected macrophages were quantified and treated for 48 h with quercetin 3-O-methyl ether (0, 70, 140, or 280 *μ*M) or strychnobiflavone (0, 40, 80 or 160 *μ*M) for 48 h at 24°C in 5% CO_2_. Cells were washed in RPMI 1640 and incubated with 4% paraformaldehyde for 15 min, at which time they were treated with 70% ethanol in an ice bath for 4 h and again washed three times with sterile PBS. Amphotericin B was used as a control. The percentage of the inhibition of *Leishmania* intra-macrophage viability was determined by counting 200 cells in triplicate. Data shown are representative of four independent experiments, performed in triplicate, which presented similar results.

### 2.9. Statistical Analysis

Data were analyzed using the GraphPad Prism software (version 5.0 for Windows). The difference among the groups was evaluated by the one-way ANOVA, followed by Bonferroni's post-test for multiple comparisons. Differences were considered significant when *P* < 0.05.

## 3. Results

### 3.1. Structural Elucidation of Flavonoids

**Figure 1:**
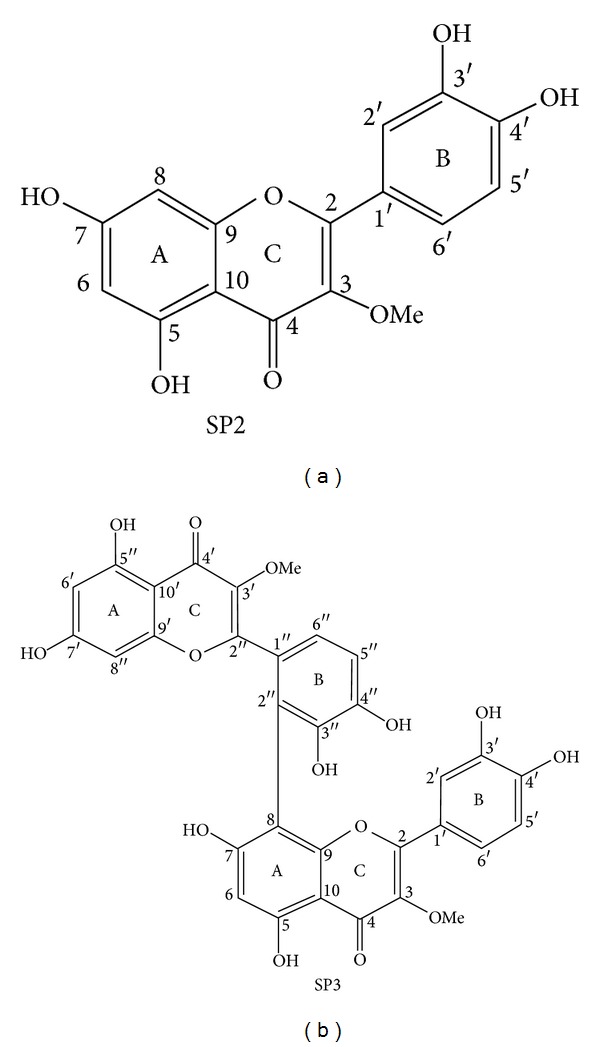
Structural characterization of quercetin 3-O-methyl ether (a) and strychnobiflavone (b).

The 29 different groups identified in a bioactivity-guided fractionation were evaluated in their antileishmanial activity, and four of them were selected. These groups were purified by recrystallization using dichloromethane, and rendered the purification of quercetin 3-O-methyl ether and strychnobiflavone. The structural and chemical characterizations of the two flavonoids isolated from *S. pseudoquina* in this study are shown in Table 1. In addition, the chemical structures of the described products are shown in Figure 1. All data obtained confirmed the identification of the two pure compounds present in the four selected fractions, which exhibited an effective antileishmanial activity (Table 1 and Figure 1). In addition, the structural identification of quercetin 3-O-methyl ether and strychnobiflavone were compared with literature records in order to prove to be identical compounds [20, 22].

### 3.2. Antileishmanial Activity of the Ethyl Acetate Extract and Purified Compounds

The inhibition of *Leishmania* viability using an ethyl acetate extract obtained from  *S. pseudoquina* was evaluated against stationary-phase promastigotes of *L. amazonensis*. It could be observed that this extract was effective against parasites, presenting an IC_50_ value of 24.9 *μ*g/mL (Table 2). In this context, a bioactivity-guided fractionation was performed, and 29 different groups were obtained and individually evaluated in their antileishmanial activity, in which 4 groups were identified as presenting the best biological activity (data not shown). These groups were submitted to rigorous purification processes using NMR, and 3-O-methyl ether and strychnobiflavone identified the levels of antileishmanial activity. As such, these two compounds were individually evaluated in their antileishmanial activity against promastigotes of* L. amazonensis*, where the IC_50_ values were 8.08 *μ*M and 3.16 *μ*M, respectively (Table 2). Amphotericin B was used as a control. 

### 3.3. Cytotoxicity and Hemolytic Activity

The cytotoxicity of the ethyl acetate extract, 3-O-methyl ether, and strychnobiflavone was investigated using murine macrophages. The *in vitro* assay demonstrated no significant toxicity in cells after incubation with these products. The values of CC_50_ were of 258 *μ*g/mL, 199 *μ*M, and 125 *μ*M, respectively, and the calculated selectivity index (SI) was 10.4, 24.62, and 39.55, respectively (Table 2). The hemolytic activity in O^+^ human red blood cells was also determined to be an additional cytotoxic parameter, and no significant damage to human erythrocytes could be observed after incubation with the ethyl acetate extract, quercetin 3-O-methyl ether, or strychnobiflavone products (Table 2).

### 3.4. Inhibition of Infection and Treatment of Infected Macrophages

The *Leishmania* infectivity treated with quercetin 3-O-methyl ether or strychnobiflavone was also evaluated in murine macrophages. For this purpose, *L. amazonensis *were preincubated with quercetin 3-O-methyl ether (0, 70, 140, or 280 *μ*M) or strychnobiflavone (0, 40, 80, or 160 *μ*M) for 4 h at 24°C. These parasites were able to infect 46.1% and 23.8% of the macrophages, respectively, while parasites that were not preincubated with the products were able to infect about 87% of the macrophages. In this context, parasites presented reductions in their infectivity in the order of 47% and 72.6% after treatment with quercetin 3-O-methyl ether or strychnobiflavone, respectively (Table 3). An evaluation of the capacity of the quercetin 3-O-methyl ether and strychnobiflavone in treating macrophages previously infected with *L. amazonensis* was performed. Cells were preinfected with promastigotes of *L. amazonensis *(in a ratio of 10 parasites per 1 macrophage) and later treated with 70, 140, or 280 *μ*M of quercetin 3-O-methyl ether, or with 40, 80, or 160 *μ*M of strychnobiflavone, for 48 h at 24°C and 5% CO_2_. The results shown that macrophages that were infected and later treated with quercetin 3-O-methyl ether or strychnobiflavone presented reductions in the parasite number in the order of 40% and 87%, respectively (Table 4).

## 4. Discussion

Plants of the genus *Strychnos* are known for their bioactivity in folk medicine. Nevertheless, few studies have been performed to evaluate their biological activity or to identify and characterize the compounds responsible for this effect. *Strychnos pseudoquina* is a plant used in Brazilian folk medicine in the treatment of diseases, such as malaria [17]. One previous study demonstrated the activity of a methanolic extract obtained from the leaves of *S. pseudoquina* in protecting mice against injuries to the gastric mucosa caused by nonsteroidal anti-inflammatory drugs [25]. Phytochemical studies developed by Monache et al. [26] and Nicoletti et al. [20] led to the biochemical isolation of bisnordihydrotoxiferine, diaboline, and 11-methoxydiaboline alkaloids purified from *S. pseudoquina* leave extracts to show certain biological activities. 

**Figure 2:**
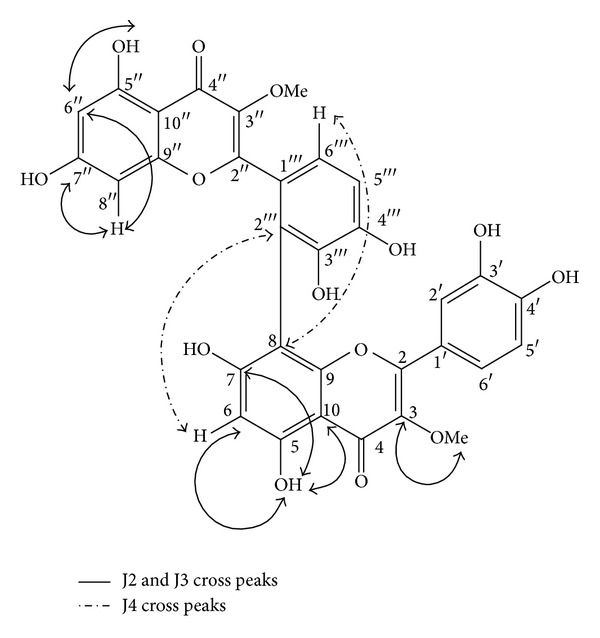
Key HMBC correlations of strychnobiflavone.

In this context, only limited data are available in the literature concerning the biological properties and chemical composition of stem bark extracts obtained from  *S. pseudoquina*, and in relation to the identification of the pure compounds of this plant, that may be responsible for these biological effects. In this light, the purpose of this study was to evaluate the antileishmanial activity of an ethyl acetate extract obtained from *S. pseudoquina *and to perform the bioactivity-guided fractionation in an attempt to identify the active and isolate components. This strategy led to the isolation of two flavonoids after a rigorous purification process by adsorption chromatography and recrystallization. These isolated compounds were the main factors responsible for the antileishmanial activity observed in the ethyl acetate extract evaluated in this study. Therefore, the obtained spectroscopic data performed here, and their comparison with assignments reported for quercetin 3-O-methyl ether and strychnobiflavone led to a unique proposal of identification of structures of the two isolated flavonoids. 2D NMR techniques, in particular HMBC (Figure 2), allow us to unambiguously establish the structural assignment of strychnobiflavone without requiring chemical modification, as used by Nicoletti et al. [20].

Natural compounds are an important source of new and selective agents for the treatment of tropical diseases caused by protozoans, such as leishmaniasis [27–29]. The antileishmanial activity observed in total extracts prepared from these materials has been attributed to compounds belonging to diverse chemical groups, such as isoquinoline alkaloids, indole alkaloids, quinones, terpenes, steroids, carbohydrates, lignans, proteins, and flavonoids [28, 30–32]. In the present study, the quercetin 3-O-methyl ether and strychnobiflavone proved to be responsible for the antileishmanial activity observed in the ethyl acetate extract of *S. pseudoquina* against *L. amazonensis*. Results shown that the quercetin 3-O-methyl ether presented an IC_50_ value in the order of 8.08 *μ*M, while the strychnobiflavone presented an IC_50_ value of 3.16 *μ*M. Previous reports have shown that flavonoids are known to have anti-inflammatory activity in mammalian cells, and their action in inflammatory responses has been attributed to an antioxidant effect [33–35]. Tasdemir et al. [36] demonstrated an antileishmanial and antitrypanosomal activities of flavonoids that had been purified from natural compounds, as well as by their analogues in *in vitro *and* in vivo *experiments. In addition, Marín et al. [31] shown that flavonoids purified from *Consolida oliveriana* were able to activate macrophages and were highly effective in *in vitro* experiments against *L. peruviana*.

Both quercetin 3-O-methyl ether and strychnobiflavone shown an effective leishmanicidal activity against *in vitro* promastigotes and amastigotes of *L. amazonensis* and proved to be effective in inhibiting the infection of phagocytic cells by parasites that had been preincubated with them, as well as in reducing the parasite burden in first infected macrophages, which were later treated with the products. In addition, the two substances presented a low cytotoxicity in murine macrophages and a null hemolytic activity in human red blood cells. Recently, da Silva et al. [18] shown that a methanolic extract prepared from *S. pseudoquina* was unable to induce toxicity in BALB/c mice. In this study, no signal or symptom of toxicity was observed in the treated cells [18]. There were no significant differences in organ weight, in water or food intake, or in the amount of faces produced by treated and control mice. On the other hand, amphotericin B proved to be quite efficient in eliminating parasites *in vitro* but was highly cytotoxic in mammalian cells, thus demonstrating some of its reported adverse effects in the patients [18].

Results presented here shown that quercetin 3-O-methyl ether and strychnobiflavone, purified from an ethyl acetate extract of *S. pseudoquina *stem bark, were more potent in combating *Leishmania* in *in vitro* experiments, as compared with an ethyl acetate extract of this plant. Since these compounds are present in relatively high concentrations in *S. pseudoquina* stem bark, the cultivation or sustainable management of this plant species may become viable for the commercial production of these flavonoids, representing an alternative to the sustainable use of the Brazilian savanna biome.

## 5. Conclusion

The results presented in this study shown the identification and first use of antileishmanial and atoxic products, based on two flavonoids purified from a common Brazilian medicinal plant. Further studies are in progress to evaluate the activity of quercetin 3-O-methyl ether and strychnobiflavone in treating murine models experimentally infected with *L. amazonensis*.

## Figures and Tables

**Table 1:** Structural and chemical characterizations of the two flavonoids purified from *S. pseudoquina*. ^1^H and ^13^C chemical shifts (ppm) obtained for quercetin 3-O-methyl ether and strychnobiflavone.

Positions	Quercetin 3*-*O-methyl ether (*δ*_H_)^a^	Quercetin 3*-*O-methyl ether (*δ*_H_)^c^	Quercetin 3*-*O-methyl ether (*δ*_C_)^a^	Quercetin 3*-*O*-*methyl ether (*δ*_C_)^c^	Strychnobiflavone (*δ*_H_)^b^	Strychnobiflavone (*δ*_H_)^d^	Strychnobiflavone (*δ*_C_)^b^	Strychnobiflavone (*δ*_C_)^d^
2	—	**—**	158.5^e^	157.3	—	**—**	155.5	157.4
3	—	**—**	139.7	138.4	—	**—**	137.4	138.7
4	—	**—**	180.1	178.9	—	**—**	178.0	179.1
5	—	**—**	166.0	161.9	—	**—**	154.1	162.0
6	6.18; d (*J* = 2.0 Hz)	6.21	99.9	98.6	6.16; s	6.26; s	97.9	98.8
7	—	—	163.2	164.8	—	—	159.9	164.2
8	6.37; d (*J* = 2.0 Hz)	6.41	94.8	93.5	—	—	102.8	103.6
9	—	—	158.1^e^	156.8	—	—	161.2	155.5
10	—	—	106.0	104.7	—	—	104.0	105.5
1′	—	—	123.6	121.8	—	—	121.1	123.0
2′	7,61; d (*J* = 2.0 Hz)	7.64	116.5^f^	115.3	7.44; d (*J* = 2.0 Hz)	7.60; d (*J* = 2 Hz)	115.9	124.0
3′	—	—	146.6	145.3	—	—	145.1	148.7
4′	—	—	150.1	148.8	—	—	148.6	145.3
5′	6,89; d (*J* = 8.4 Hz)	6.92	116.6^f^	115.2	6.71; d (*J* = 8.4 Hz)	6.82; d (*J* = 8 Hz)	115.4	115.7
6′	7,51; dd (*J* = 8.4 Hz; 2.0 Hz)	7.55	122.5	121.2	7.05; dd (*J* = 8.4 Hz; 2.0 Hz)	7.29; dd (*J* = 8 Hz; 2 Hz)	120.2	121.7
2′′	—	—	—	—	—	—	159.1	157.4
3′′	—	—	—	—	—	—	138.2	138.7
4′′	—	—	—	—	—	—	177.8	179.0
5′′	—	—	—	—	—	—	156.3	161.9
6′′	—	—	—	—	6.07; d (*J* = 1.6 Hz)	6.10 (*J* = 2 Hz)	98.4	98.9
7′′	—	—	—	—	—	—	163.9	162.5
8′′	—	—	—	—	5.66; d (*J* = 1.6 Hz)	5.84; d (*J* = 2 Hz)	92.8	93.6
9′′	—	—	—	—	—	—	161.2	156.4
10′′	—	—	—	—	—	—	104.2	105.5
1′′′	—	—	—	—	—	—	122.3	122.9
2′′′	—	—	—	—	—	—	119.7	116.2
3′′′	—	—	—	—	—	—	144.2	147.9
4′′′	—	—	—	—	—	—	147.5	148.8
5′′′	—	—	—	—	6.97; d (*J* = 8.4 Hz)	7-7.1; m	114.1	114.9
6′′′	—	—	—	—	7.05; d (*J* = 8.4 Hz)	7-7.1; m	121.4	119.8
3*-*O*-*Me	3.77; s	3.80	60.6	59.35	3.79; s	3.82; s	59.8	60.3
3′′*-*O*-*Me	—	—	—	—	3.43; s	3.63; s	59.6	59.9
5-OH	12.47; s	—	—	—	12.68; s	12.8; s	—	—
5′′-OH	—	—	—	—	12.56; s	12.6; s	—	—

^a^CD_3_OD; ^b^DMSO-*d*_6_; ^c^DMSO-*d*_6_ (Guinot et al. 2009 [22]); ^d^acetone-*d*_6_ (Nicoletti et al. 1984 [20]); ^e,f^interchangeable.

**Table 2:** Antileishmanial activity, cytotoxicity, and selective index found for the ethyl acetate extract of *S. pseudoquina*, quercetin 3-methyl ether, and strychnobiflavone.

Compounds	IC_50_^a^	CC_50_^b^	RBC_50_^c^	SI^d^
Ethyl acetate extract (*μ*g/mL)	24.9 ± 5.2	257.9 ± 35.9	180.8 ± 9.9	10.4
Quercetin 3-methyl ether (*μ*M)	8.1 ± 1.5	199.0 ± 25.9	155.0 ± 33.2	24.6
Strychnobiflavone (*μ*M)	3.2 ± 0.2	125 ± 4.5	209.6 ± 2.5	39.6
AmpB (*μ*M)	0.1 ± 0.13	0.8 ± 0.2	ND^e^	9.9

The results are expressed as medium ± standard deviation. ^a^Value of inhibitory concentration of 50% of promastigotes of *L. amazonensis*. ^b^Value of inhibitory concentration of 50% of murine macrophages. ^c^Value of 50% of human red blood cell lysis. ^d^Selectivity Index (ratio between CC_50_ and IC_50_). ^e^Not Done. AmpB (amphotericin B) was used as a control drug. Values are the average of four independent experiments, which presented similar results.

**Table 3:** Infection of macrophages using pre-treated promastigotes of* Leishmania amazonensis*.

Compounds	Concentration	Percentage of infected macrophages after treatment/number of amastigotes per cell^a^
Ethyl acetate extract (*μ*g/mL)	100	47.5/2.6
	50	54.5/2.8
	25	63.1/7.1
	0	87.0/8.4

Quercetin 3-O-methyl ether (*μ*M)	280	46.1/2.4
	140	61.9/3.9
	70	67.8/4.5
	0	87.0/8.4

Strychnobiflavone (*μ*M)	160	23.8/1.2
	80	47.8/2.2
	40	53.0/3.0
	0	87.0/8.4

^a^The percentage of infected macrophages and the number of amastigotes per cell were determined by counting 200 cell coverslips, performed in triplicate. Values are representative from four independent experiments, which presented similar results.

**Table 4:** Treatment of infected macrophages.

Products	Concentration	Percentage of infected macrophages after treatment/number of amastigotes per cell^a^
Ethyl acetate extract (*μ*g/mL)	100	48.9/1.7
	50	50.0/1.8
	25	70.3/4.4
	0	87.0/8.4

Quercetin 3-O-methyl ether (*μ*M)	280	46.3/1.7
	140	68.0/ 4.4
	70	72.4/5.6
	0	87.0/8.4

Strychnobiflavone (*μ*M)	160	9.6/0.4
	80	26.3/1.3
	40	37.9/1.5
	0	87.0/8.4

^a^The percentage of infected macrophages and the number of amastigotes per cell were determined by counting 200 cell coverslips, performed in triplicate. Values are representative from four independent experiments, which presented similar results.
